# Occupational Exposure to Dromedaries and Risk for MERS-CoV Infection, Qatar, 2013–2014

**DOI:** 10.3201/eid2108.150481

**Published:** 2015-08

**Authors:** Chantal B.E.M. Reusken, Elmoubasher A.B.A. Farag, Bart L. Haagmans, Khaled A. Mohran, Gert-Jan Godeke, Stalin Raj, Farhoud Alhajri, Salih A. Al-Marri, Hamad E. Al-Romaihi, Mohamed Al-Thani, Berend-Jan Bosch, Annemiek A. van der Eijk, Ahmed M. El-Sayed, Adel K. Ibrahim, N. Al-Molawi, Marcel A. Müller, Syed K. Pasha, Christian Drosten, Mohd M. AlHajri, Marion P.G. Koopmans

**Affiliations:** Erasmus Medical Center, Rotterdam, the Netherlands (C.B.E.M. Reusken, B.L. Haagmans, V.S. Raj, A.A. van der Eijk, M.P.G. Koopmans);; Supreme Council of Health, Doha, Qatar (E.A.B.A Farag, S.A. Al-Marri, H.E. Al-Romaihi, M. Al-Thani, A.M. El-Sayed, M.M. AlHajri);; Agricultural Research Center, Cairo, Egypt (K.A. Mohran);; Ministry of Environment, Doha (K.A. Mohran, F. Alhajri);; National Institute for Public Health and the Environment, Bilthoven, the Netherlands (G.-J. Godeke, M.P.G. Koopmans);; Utrecht University, Utrecht, the Netherlands (B.-J. Bosch);; Leawaina Camel Hospital, Dukhan, Qatar (A.K. Ibrahim, S.K. Pasha);; Hamad Medical Centre, Doha (N. Al-Molawi);; University of Bonn Medical Center, Bonn, Germany (M.M. Müller, C. Drosten)

**Keywords:** Middle East respiratory syndrome coronavirus, MERS-CoV, coronavirus, MERS, zoonoses, camels, dromedaries, transmission, infectious, Qatar, epidemiology, viruses, exposure, contact, risk

## Abstract

We determined the presence of neutralizing antibodies to Middle East respiratory syndrome coronavirus in persons in Qatar with and without dromedary contact. Antibodies were only detected in those with contact, suggesting dromedary exposure as a risk factor for infection. Findings also showed evidence for substantial underestimation of the infection in populations at risk in Qatar.

Since Middle East respiratory syndrome coronavirus (MERS-CoV) was first detected in 2012, approximately 1,000 human infections have been reported to the World Health Organization, all linked to residence in or travel to countries on the Arabian Peninsula (*1*). Dromedaries (*Camelus dromedarius*) are thought to play a central role in MERS epidemiology because widespread evidence of MERS-CoV–specific antibodies and virus shedding in camels was found (*2*), and highly similar viruses have been detected in humans and dromedaries at the same location (*3*,*4*). These data suggest a direct zoonotic risk for MERS-CoV infection among persons in contact with camels. We describe a comparative serologic investigation in Qatar among persons with and without daily occupational exposure to dromedaries.

## The Study

We used 498 anonymized serum samples from persons in Qatar with and without dromedary contact (Technical Appendix) and control serum from Europe (National Institute for Public Health and the Environment, Bilthoven, the Netherlands; and University of Bonn, Bonn, Germany). Sampling in Qatar was cleared by the Ethics and Institutional Animal Care and Use Committees of the Medical Research Center, Hamad Medical Corporation (permit 2014-01-001). Samples from the Netherlands were used in accordance with the Dutch Federation of Medical Scientific Associations’ code of conduct for proper use of human tissue. Samples from Germany were used in accordance with German national laws.

Of the 498 samples, 294 were from persons with daily occupational contact with dromedaries (cohorts A–D) and 204 were from persons without camel contact (cohorts E–G). Cohort A consisted of 109 healthy workers (5 camel slaughterers [subcohort A1] and 104 sheep slaughterers [A2]) at the central slaughterhouse in Doha, Qatar. All workers lived together and had contact with camels and sheep at the central animal market (CAM). Cohort B consisted of 8 CAM workers. Cohort C consisted of 22 healthy men living and working at the Al Shahaniya barn complex near the international dromedary racing track, and cohort D consisted of 155 healthy men living and working on a dromedary farm in Dukhan, western Qatar; molecular data showed ongoing circulation of MERS-CoV in dromedaries in these locations (Technical Appendix). Cohort E consisted of 56 random samples from construction workers in Qatar. Cohort F consisted of 10 samples from persons working and living at a complex with 200 sheep barns in northern Qatar. Cohort G consisted of 138 samples for confirming specificity of the testing algorithm (66 samples from the Netherlands and Germany from persons with recent human CoV infection [subcohort G1] and 72 samples from the Netherlands obtained for routine testing from persons with suspected *Bordetella pertussis* infection [G2]).

We used microarray technology as described (*3*,*5*,*6*) to analyze samples for the presence of IgG reactive with MERS-CoV S1 antigen (Table). To avoid overinterpretation of data, we set the reactivity cutoff at 30,000 relative fluorescent units for subsequent analyses (*6*). Samples from 20 of 294 persons with camel contact were reactive; no control or noncontact samples were reactive. Among camel handlers at the Al Shahaniya and Dukhan locations, 4 of 22 and 8 of 155, respectively, had antibodies to MERS-CoV S1. At the CAM, 1 of 8 handlers had antibodies. At the slaughterhouse location, 3 of 104 sheep slaughterers and 4 of 5 camel slaughterers were antibody-positive (Figure). 

**Table:** Results of MERS-CoV serologic testing of humans with and without dromedary contact, Qatar, 2013–2014*

Exposure type, cohort	Country	Serum samples tested by		
		S1 assay, no. positive/no. tested	PRNT_90_, no. positive/no. tested†	
			S1-positive	S1-negative
Dromedary contact		20/294	10/20	1/35
A, slaughterhouse workers				
A1, camel slaughterers	Qatar	4/5	2/4 (40, 20)	NT
A2, sheep slaughterers (contact with camels/camel slaughterers)	Qatar	3/104	2/3 (20, 20)	1/16 (20)
B, central animal market workers	Qatar	1/8	0	NT
C, barn workers at international camel racing track	Qatar	4/22	3/4 (40, 40, 20)	NT
D, camel farm workers	Qatar	8/155	3/8 (40, 40, 20)	0/19
No dromedary contact		0/204	NA	0/48
E, construction workers	Qatar	0/56	NA	0/48
F, sheep farmers	Qatar	0/10	NA	NT
G, specificity controls				
G1, recent infection with a common hCoV	GER, NL	0/66	NA	NT
G2, suspected infection with *Bordetella pertussis*	NL	0/72	NA	NT

*GER, Germany; hCoV, human coronavirus; MERS-CoV, Middle East respiratory syndrome coronavirus; NA, not applicable; NL, the Netherlands; NT, not tested; PRNT_90_, 90% plaque-reduction neutralization test; S1, MERS-CoV S1 antigen. †Nos. in parentheses are reciprocal antibody titers in PRNT_90_.

**Figure:**
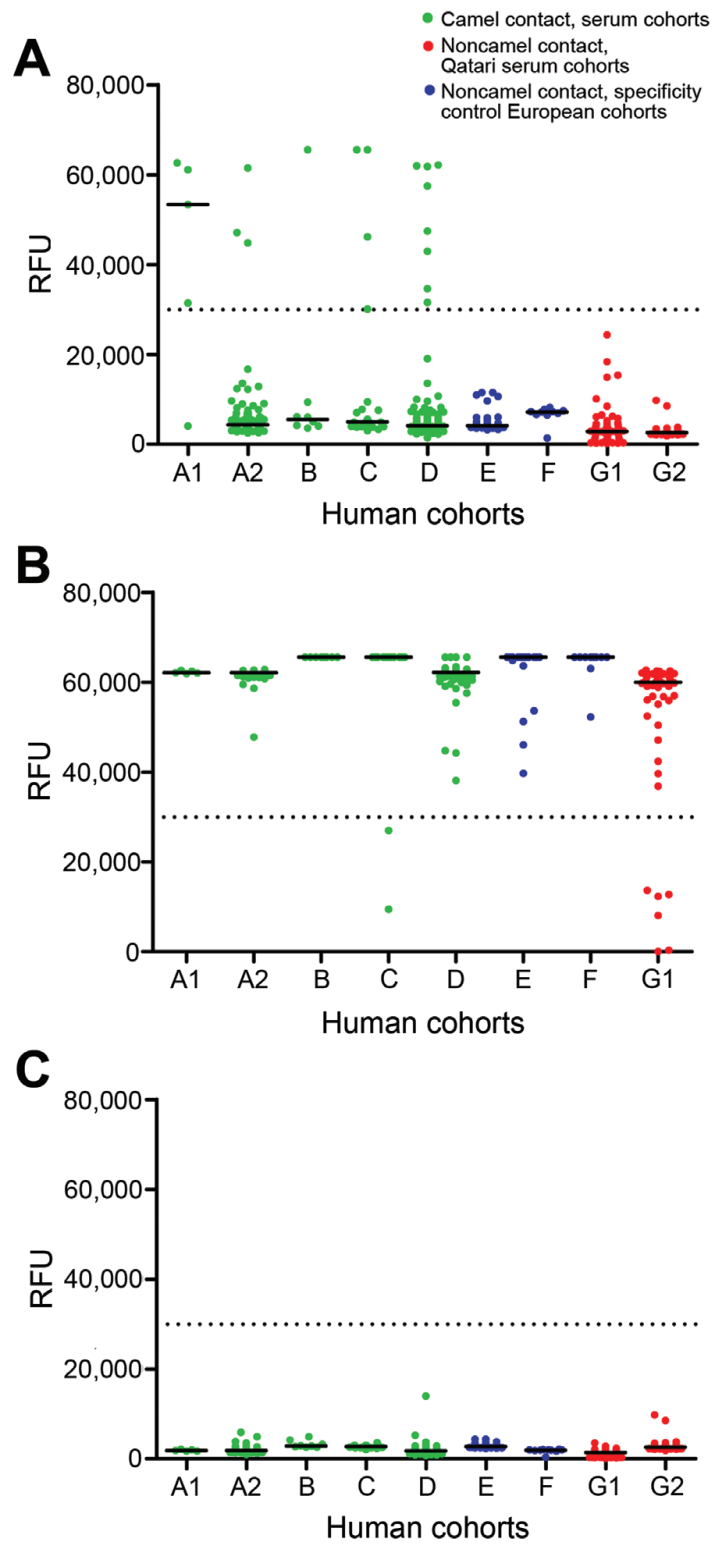
Reactivity of human serum samples, from persons with and without dromedary contact, with S1 antigens of various coronaviruses (CoVs), Qatar, 2013–2014. A) Middle East respiratory syndrome CoV S1; B) human CoV OC43 S1; C) severe acute respiratory syndrome CoV S1. Relative fluorescent units (RFU) are shown at a serum dilution of 1:20. Black lines indicate median; dotted black lines at 30,000 RFU depict cutoff for analysis. Human cohorts: A1, camel slaughterers; A2, sheep slaughterers who had contact with dromedaries and camel slaughterers; B, workers at the central animal market; C, barn workers at the international camel racing track; D, workers on camel farms; E, construction workers; F, sheep farmers; G1, persons recently infected with a common human CoV (serum samples from the Netherlands and Germany); G2, persons with suspected *Bordetella pertussis* infection (serum samples from the Netherlands).

Samples from subcohort G1 (n = 66) and from all camel-contact cohorts were tested for antibodies to CoV OC43 S1, a common human CoV; all showed high seropositivity (range 89%–100%) (Figure). All 498 samples were tested for reactivity to severe acute respiratory syndrome CoV S1; none reacted (Figure).

We used a 90% plaque-reduction neutralization test (PRNT_90_) to confirm the presence of MERS-CoV–specific antibodies in serum samples from camel handlers. For testing, we used the 20 samples that were reactive to MERS-CoV S1 and a random selection of nonreactive samples from camel-contact (n = 35) and noncontact (n = 48) cohorts. Results were positive for 10 of the 20 MERS-CoV S1 antibody–positive samples (reciprocal titers of 20 or 40) (Table).

All but 1 of the 35 samples from persons with camel contact who had negative S1 ELISA screening results were negative by PRNT_90_; the positive sample had a reciprocal titer of 20 (Table). All 48 samples from the noncontact cohorts were negative by PRNT_90_. This finding may indicate an underestimation of MERS-CoV seroprevalence by S1 testing. Furthermore, 6 samples from S1-positive and 2 from S1-negative persons with camel contact showed a reciprocal titer of 10, but titers of 10 were not observed in the noncontact cohorts. Five of these 8 reactive samples were also positive in a whole-virus MERS-CoV immunofluorescence assay at dilution 1:100; however, we regarded these as negative to avoid overinterpretation of data (data not shown).

## Conclusions

We detected MERS-CoV neutralizing antibodies in healthy persons who had daily occupational contact with dromedaries but not in persons without such contact. Only limited evidence is available regarding the presence of MERS-CoV antibodies in the general human population or in specific population cohorts. However, an overall seroprevalence of 0.15% was found in a cross-sectional study in Saudi Arabia, and among slaughterhouse workers, neutralizing antibodies were detected in 5 of 140 participants (*7*). This finding is similar to our finding among slaughterhouse workers: 7 of 109 were MERS-CoV antibody–positive. Four other studies lacked serologic evidence of MERS-CoV infection in humans with occupational exposure to dromedaries (*8*–*11*). However, only 1 of those studies documented actual MERS-CoV circulation in dromedaries during human contact, and it was concluded that MERS-CoV was not highly transmissible from camels to humans, although only 7 persons had regular contact with only 1 herd (*8*). On several occasions, the percentage of camels shedding MERS-CoV was high (60%) at the CAM and slaughterhouse (C.B.E.M. Reusken, unpub. data). Thus, locations with a continuous flow of dromedaries with different places of origin and different immune statuses may enable prolonged circulation of MERS-CoV and sustained exposure of dromedary handlers to the virus; in Qatar, such locations would include the CAM, slaughterhouse, and barns near the international racing tracks. 

In this study, PRNT_90_-derived antibody titers were relatively low compared with those from earlier studies of MERS patients and dromedaries (*2*; B.L. Haagmans, unpub. data)*.* The lower titers might reflect the apparent asymptomatic manifestation of MERS-CoV infection, individual differences in susceptibility, or both (*2*). Also, primary infections may result in a short-lived antibody peak followed by a rapid waning of antibody, depending on virus and host properties (*12*), as seen in influenza A(H5N1) virus infection: antibody levels are higher in symptomatic than asymptomatic H5N1-infected persons, and antibodies wane more quickly during asymptomatic infection (*13*). MERS-CoV antibody kinetics and the persistence of antibodies detected by different serologic methods are not known. Such parameters are needed to estimate the force of infection on the basis of serologic data (*14*).

MERS-CoV–seropositive participants in this study did not report severe health problems, giving evidence for frequent unrecognized human infections. Assuming the health histories are accurate, this finding implies that the current overall MERS-CoV–associated death rate of 37.1% (*1*) is most likely an overestimation of the actual rate and that most infections may be asymptomatic or mild. A major issue to be resolved is whether, and to what extent, asymptomatic cases contribute to the spread of MERS-CoV; it is well recognized that variability in disease transmission exists among humans (*15*).

**Technical Appendix.** Description of human cohorts for serum samples.
