# Supplementary material for: Occupational Exposure to Dromedaries and Risk for MERS-CoV Infection, Qatar, 2013–2014
Source: Emerg Infect Dis. 2015 Aug;21(8):1422–5. doi: 10.3201/eid2108.150481 (PMC4517733; doi:10.3201/eid2108.150481)
Supplement: Supplementary file 1 — Technical Appendix. Description of human cohorts for serum samples. [file 15-0481-Techapp-s1.pdf]

Article DOI: http://dx.doi.org/10.3201/eid2108.150481

Occupational Exposure to Dromedaries and Risk for MERS-CoV Infection, Qatar, 2013–2014

Technical Appendix

Description of Human Serum Cohorts

The human serum cohorts consisted of the following:

A) Anonymized serum samples from 109 healthy males (immigrants) collected in 2014 and working at the central slaughterhouse in Doha, Qatar. Only five workers exclusively work in camel slaughter in Qatar, with 3–20 years of experience (A1). All five were sampled. The other workers exclusively slaughter sheep (A2). However, all workers live together and have contact with the animals (camels, sheep) at the central animal market. The percentage of camels presented for slaughter that shed MERS-CoV was high at several occasions (*1*) (C.B.E.M. Reusken, unpub. data).

B) Anonymized sera from eight healthy males (originally from India, Nepal, Pakistan and Sudan) working at the Central animal market and collected in 2014. The market serves mainly camels and sheep but goats, cows, horses and donkeys as well. The market comprises ≈100 pens for camels, with ≈20 animals per pen. The camels originate from Australia, Iran, Oman, Pakistan, Qatar, Saudi-Arabia, Somalia, Sudan and Syria, and are sold for slaughter or use (pet, breeding, milk production) in Qatar. Animals introduced from Australia are presumed to be naive for MERS-CoV (*2*). This cohort has daily close contact with animals and their secretions.

C) Serum samples from 22 healthy males living and working at the Al Shahaniya barn complex near the Qatar international dromedary racing track. During the racing season from October through March, camels from around the Gulf area visit and stay together with Qatari camels at the Al Shahaniya barn complex. The barn complex consists of 749 barns with an estimated total of 14,000 camels. These persons originate from India, Nepal, Pakistan or Sudan and have daily close contact (nurturing, cleaning, grooming, training for racing) with camels residing at the barns. The sera were collected in 2013 and 2014. Published and

unpublished molecular data of camel samples taken in the period 2012–2014 and the connection of the first two human cases in Qatar in 2015 with this barn complex show ongoing circulation of MERS-CoV in the complex (*3*–*6*).

D) Serum samples from 155 healthy males living and working at a camel farm in the Dukhan area, West Qatar, collected in 2013 and 2014. The farm consists of milking, breeding and racing herds with an estimated 6.000 camels (*4*). The camel handlers originated from India, Nepal, Pakistan or Sudan, were 20–35 years of age and had daily, intensive contact with camels (nurturing, cleaning, grooming, veterinary care, training for races). The herds at the Dukhan farm showed molecular evidence for circulation of MERS-CoV at several occasions during 2013 and 2014 (A.K. Ibrahim, unpub. data).

E) Random, anonymized serum samples collected in 2014 from 56 males working for construction companies (laborers, metal workers, guards, plumbers, crane operators, drivers) and living in company camps in Doha, Qatar. All workers were healthy when samples were collected. The workers were <35 years of age and of Asian origin. There is no occupational contact with dromedaries, no ownership of dromedaries.

F) Anonymized serum samples from ten healthy males (immigrants) working and living at a complex with 200 sheep barns in North Qatar. At the barns >95% of the animals are sheep, other animals are goats, chickens, ducks. There is no contact with camels. Samples were collected in 2014.

G) Control group for specificity of the testing algorithm. G1) Anonymized serum samples from patients with a recent common human coronavirus (hCoV) infection (n = 66). Serum samples of 10 children, ages ranging from 9–14 months (2x hCoV-HKU1, 2x hCoV-OC43, 3x hCoV-229E and 3x hCoV-NL63 IgG positive sera) and obtained in 2001 in the Netherlands. Four anonymized hCoV-OC43 PCR positive sera from adults obtained in Germany in 2013. Fifty-four anonymized serum samples from adults obtained in Erasmus MC, the Netherlands in the period 2010–2014 and taken >2wks-<1 year after a respiratory tract sample tested positive for hCoVs using real-time RT-PCR technology (23x hCoV-OC43, 16x hCoV-229E, 15x hCoV-NL63. Serum had been collected at a later stage during hospitalization and subsequent routine visits to the out-patient clinic, the majority of these patients had recurrent health problems due to immune-deficiency, and was stored at Erasmus MC at −20°C. The study was approved by the local medical ethical committee (MEC approval: 2014–414). G2) Anonymized serum samples from 72 persons ranging in age from

0.1 year to 95.3 years sampled during 2008 for routine *Bordetella pertussis* serology in the Netherlands. This serum set represents a cohort biased toward patients with non-influenza–like respiratory symptoms (*7*).

References.

1. Raj VS, Farag EA, Reusken CB, Lamers MM, Pas SD, Voermans J, et al. Isolation of MERS coronavirus from a dromedary camel, Qatar, 2014. Emerg Infect Dis. 2014;20:1339–42. PubMed http://dx.doi.org/10.3201/eid2008.140663
2. Hemida MG, Perera RA, Al Jassim RA, Kayali G, Siu LY, Wang P, et al. Seroepidemiology of Middle East respiratory syndrome (MERS) coronavirus in Saudi Arabia (1993) and Australia (2014) and characterisation of assay specificity. Euro Surveill. 2014;19:pii: 20828. PubMed
3. Haagmans BL, Al Dhahiry SH, Reusken CB, Raj VS, Galiano M, Myers R, et al. Middle East respiratory syndrome coronavirus in dromedary camels: an outbreak investigation. Lancet Infect Dis. 2014;14:140–5. PubMed http://dx.doi.org/10.1016/S1473-3099(13)70690-X
4. Reusken CB, Farag EA, Jonges M, Godeke GJ, El-Sayed AM, Pas SD, et al. Middle East respiratory syndrome coronavirus (MERS-CoV) RNA and neutralising antibodies in milk collected according to local customs from dromedary camels, Qatar, April 2014. Euro Surveill. 2014;19:pii: 20829. PubMed
5. World Health Organization. Disease outbreak news; 11 Feb 2015. Middle East respiratory syndrome coronavirus (MERS-CoV) – Qatar [cited 2015 19 March 19]. http://www.who.int/csr/don/11-february-2015-mers-qatar/en/
6. World Health Organization. Disease outbreak news; 11 Mar 2015. Middle East respiratory syndrome coronavirus (MERS-CoV)–Qatar [cited 2015 Mar 19]. http://www.who.int/csr/don/11-march-2015-mers-qatar/en/.
7. Reusken C, Mou H, Godeke GJ, van der Hoek L, Meyer B, Müller MA, et al. Specific serology for emerging human coronaviruses by protein microarray. Euro Surveill. 2013;18:pii: 20441. PubMed
